# Different prognosis for stage IIB cervical cancer patients with unilateral or bilateral parametrial invasion treated with concurrent chemoradiotherapy

**DOI:** 10.1093/oncolo/oyaf329

**Published:** 2025-10-16

**Authors:** Xi-Lin Yang, Li-Chun Wei, Jian-Li He, Tie-Jun Wang, Li Ran, Li-Juan Zou, Xiao-Ge Sun, Xiao-Mei Li, Zi Liu, Yong-Gang Shi, Sha Li, Feng-Ju Zhao, Kun Gao, Wei Zhong, Guang-Hui Cheng, Ya-Li Gao, Bao-Sheng Sun, Jun-Fang Yan, Fu-Quan Zhang

**Affiliations:** Department of Radiation Oncology, Peking Union Medical College Hospital, Chinese Academy of Medical Sciences & Peking Union Medical College, Beijing 10000, China; Department of Radiation Oncology, State Key Laboratory of Complex Severe and Rare Diseases, Peking Union Medical College Hospital, Chinese Academy of Medical Sciences & Peking Union Medical College, Beijing 10000, China; Department of Radiation Oncology, Xijing Hospital, Air Force Medical University, Xi’an, Shanxi 710000, China; Department of Radiation Oncology, General Hospital of Ningxia Medical University, Yinchuan, Ningxia 750000, China; Department of Radiation Oncology, The Second Affiliated Hospital of Jilin University, Changchun 130041, China; Department of Oncology, Affiliated Cancer Hospital of Guizhou Medical University, Gui Yang, GuiZhou 550004, China; Department of Radiation Oncology, The Second Affiliated Hospital of Dalian Medical University, Liaoning 116000, China; Department of Radiation Oncology, Affiliated Hospital of Inner Mongolia Medical University, Hohhot, Inner Mongolia 010000, China; Department of Radiation Oncology, Peking University First Hospital 100000, China; Department of Radiation Oncology, First Affiliated Hospital of Xian Jiaotong University, Xi’an 719000, China; Department of Radiation Oncology, The First Affiliated Hospital of Zhengzhou University, Zhengzhou 450000, China; Department of Radiation Oncology, The 940th Hospital of Joint Logistics Support Force of Chinese People’s Liberation Army, Lanzhou 730070, China; Department of Radiotherapy, Gansu Province Cancer Hospital, Lanzhou 730050, China; Department of Gynecologic Oncology, Guangxi Medical University Cancer Hospital, 71 Hedi Road, Qingxiu District, Nanning 530000, China; Gynaecological Oncology Radiotherapy (Inpatient Area 1), The Affiliated Cancer Hospital of Xinjiang Medical University, Urumqi 830000, China; Department of Radiation Oncology, China-Japan Union Hospital of Jilin University, Jilin 130100, China; Department of Radiation Oncology, Cangzhou Central Hospital, Cangzhou, Hebei 061000, China; Department of Radiation Oncology, Peking Union Medical College Hospital, Chinese Academy of Medical Sciences & Peking Union Medical College, Beijing 10000, China; Department of Radiation Oncology, Peking Union Medical College Hospital, Chinese Academy of Medical Sciences & Peking Union Medical College, Beijing 10000, China; Department of Radiotherapy, Jilin Province Cancer Hospital, Changchun 130000, China

**Keywords:** stage IIB, cervical cancer, unilateral parametrial invasion, bilateral parametrial invasion, SHapley Additive exPlanation

## Abstract

**Objective:**

To compare the survival difference between 2018 International Federation of Gynecology and Obstetrics (FIGO) stage IIB cervical cancer (CC) patients with unilateral parametrial invasion (UL) and bilateral parametrial invasion (BL) disease, and explore the significant role of parametrial invasion (PI) in prognosis prediction.

**Methods:**

A total of 506 stage IIB CC patients were identified from the multi-center study, and patients were divided into UL and BL groups according to gynecological and radiological examination. Survival outcomes were estimated and compared between 2 groups before and after propensity scoring matching (PSM). The role of upper 2/3 vaginal invasion (VI) in impacting survival probability was also assessed. The random forest (RF) model was constructed and validated to select important features related to survival outcomes and predict prognosis for these patients. The SHapley Additive exPlanation (SHAP) was further introduced to provide a better understanding toward the findings from the RF model.

**Results:**

Significant better 5-year overall survival (OS) was observed among patients with UL disease whether before (BL: 61.7% [95% CI: 57.0%-66.4%]; UL: 84.8% [95% CI: 82.4%-87.2%]; HR = 2.83, 95% CI: 1.90-4.20, *P* < 0.001) or after PSM (BL: 61.3% [95% CI: 56.6%-66.0%]; UL: 81.2% [95% CI: 77.3%-85.1%]; HR = 2.51, 95% CI: 1.56-4.04, *P* < 0.001). Similar findings could also be observed in terms of progression-free survival (PFS). The presence of VI didn’t significantly impair the survival probability, whether in the UL or BL group (all *P* > 0.05). RF model was constructed, which possessed decent predictive ability both in the training (area under the receiver operating characteristic curve [AUC] = 0.893; 95% CI: 0.874-0.912) and validation cohort (AUC = 0.879; 95% CI: 0.801-0.957). PI was identified to be the paramount feature in affecting the survival outcomes for stage IIB CC patients through the Beeswarm summary plot and bar chart in SHAP analysis.

**Conclusion:**

Our findings demonstrated that 2018 FIGO stage IIB CC patients with BL disease had a worse prognosis than those with UL disease, and PI was the most significant feature in prognosis prediction for these patients.

Implications for practice This study first assessed the survival differences between unilateral parametrial invasion (UL) and bilateral parametrial invasion (BL) among stage IIB cervical cancer patients undergoing definitive concurrent chemoradiotherapy (CCRT), where a machine learning algorithm was utilized to identify parametrial invasion (UL or BL) as the most important factor affecting survival probability among these patients. Specifically, patients with UL disease outperformed those with BL disease in survival outcomes. In practice, more attention should be paid to stage IIB cervical cancer patients with BL disease, and adjuvant chemotherapy might be an option for them to improve the outcomes.

## Introduction

Cervical cancer (CC) was the fourth most common cancer in women globally, with an estimated 661 021 new cases and 348 189 related deaths worldwide in 2022.^1^ Approximately 10% of CC corresponding deaths occurred in China, given the extremely large population. The decreasing trends of incidence for CC could be observed in developed countries, while in China, it showed the opposite way, with approximately 15.5% and 11.2% increase in incidence and mortality rate since 2000, respectively.^2^^,^^3^

The International Federation of Gynecology and Obstetrics (FIGO) staging system for CC was mainly based on gynecological examination before 2018 until computed tomography (CT)/magnetic resonance imaging (MRI) was incorporated and combined with clinical and surgical findings into the decision process.^4^^,^^5^ While controversy still exists on the new version, the latest FIGO staging system might be the optimal tool to differentiate the survival probability for CC patients at present.^6^^,^^7^ The stage II CC included stage IIA and IIB, where IIB disease was defined as the primary tumor invaded the parametrium without reaching the pelvic sidewall.^8^ Concurrent chemoradiotherapy (CCRT), consisting of external beam radiotherapy (EBRT) and intracavity brachytherapy (BRT) plus platinum-based chemotherapy, was recommended as the standard treatment modality for locally advanced cervical cancer (LACC), including stage IIB patients internationally or nationally.^9^^,^^10^

Historical studies have investigated the prognostic factors for stage IIB CC patients, where non-squamous histology, older age, larger tumor size, and higher systemic inflammatory indexes (SIIs) were revealed to be associated with poorer survival for these patients.^11–14^ However, only 1 previous research has evaluated the survival difference between unilateral parametrial invasion (UL) disease and bilateral parametrial invasion (BL) disease among stage IIB CC patients, which showed that BL patients had worse 4-year OS compared to UL patients.^15^ Nevertheless, it was worth reappraising the survival difference between UL and BL among stage IIB CC patients, given that this study was conducted of patients treated between 1973 and 1978 when less advanced radiation technique was used in clinical practice. Besides, patients with simultaneous LN metastasis and PI would also be included in the analysis, given that stage IIIC hasn’t been officially proposed during that period, therefore, it was imperative to study this topic with meticulous selection of the patients without LN metastasis. Therefore, a multi-center study was conducted to assess whether a survival difference existed between UL disease and BL disease among stage IIB CC patients. Furthermore, the SHapley Additive exPlanation (SHAP), an interpretable artificial intelligence framework, was introduced to better appraise the prognostic significance of UL and BL in stage IIB CC patients.

## Materials and methods

### Study population

This multicenter retrospective study was conducted in 17 tertiary medical institutions across China, and Institutional Review Board (IRB) approval was obtained from each center and the specific IRB number was listed in Supplementary Table S1. CC patients diagnosed between 2005 and 2018 in each center were reviewed and restaged according to 2018 FIGO staging system. In specific, the biggest difference between the old and 2018 version of the FIGO staging system was that IB2 was upstaged to IB3 in the new version, and IB1 in the old version was divided into IB1 and IB2 according to the tumor size. Besides, any patients with pelvic and para-aortic lymph node metastasis would be upstaged to IIIC and IIIC2, respectively.^5^

### Inclusion criteria

We enrolled CC patients with 2018 FIGO stage IIB disease who received CCRT as primary treatment in this study. Noteworthy, only patients with parametrial invasion (PI) simultaneously confirmed by gynecological examination and MRI scanning would be recognized as stage IIB CC patients in our study, which could further enhance the reliability of the stage.

### Exclusion criteria

Patients would be excluded if any of the following criteria was met: (1) pregnant patients; (2) patients were 18 years old or younger; (3) patients received surgery as primary treatment or after CCRT; (4) patients didn’t receive pre–treatment gynecological examination or MRI scanning; (5) patients with Karnofsky Performance Score (KPS) <70 or severe comorbidities; (6) patients with suspicious or positive lymph node metastasis in imaging; (7) patients with incomplete follow-up information. Of note, the detailed process for selection was listed in the Supplementary Table S2.

### Treatment

All included patients underwent CCRT consisting of EBRT and BRT concurrent with platinum-based chemotherapy, where EBRT was delivered through Three-dimensional conform radiotherapy (3D-CRT), intensity modulated radiotherapy (IMRT) or volumetric modulated arc therapy (VMAT) technique and the radiation dosage was given as 45-50.4 Gy/25-28f. During the EBRT, the whole uterus, ligaments attached to uterus, parametrial tissues, part of the vagina, and pelvic lymph node drainage basin including the common iliac, external iliac, presacral, internal iliac, and obturator. BRT would be performed after the completion of EBRT with two-dimensional (2D) or three-dimensional (3D) technique and a dosage delivered as 4-8 Gy/3-8f. Surely, the interstitial BRT was 1 kind of 3D technique. The platinum-based chemotherapy would be concurrently delivered with radiotherapy, with single agent weekly or a double agent triweekly.

### Cohort definition

This was an observational cohort study and the strengthening the reporting of observational studies in epidemiology (STROBE) guideline was followed and checked as described online (https://www.strobe-statement.org/).

### Exposure group

This was an observational cohort study, and the STROBE guideline was followed and checked.^16^ The included patients would be divided into 2 groups based on gynecological examination and MRI scanning. All the corresponding MRI scanning reports were reviewed in each center, and the reports filed by a senior radiologist who majored in gynecological oncology would be accepted or the MRI would be reviewed again by the experienced radiologist from each center. Uniform criteria was adopted to diagnose PI, as irregular linear strand-like or nodular areas of hyperintense signal was noted in the parametrium. Patients with BL disease and would be classified into the exposure group.

### Control group

Patients with UL disease and would be assigned to the control group. Similarly, the disease extension to the unilateral parametrium would be simultaneously confirmed by gynecological examination and MRI scanning reports.

### Covariates and confounders

The information on age, upper 2/3 vaginal invasion (VI) status (yes or no) given that patients with stage IIB might mixed with those with upper 2/3 VI while without lower 1/3 VI, PI status (UL or BL), side of PI (right or left), tumor size, histology (squamous or non-squamous), EBRT technique (3D-CRT, IMRT, or VMAT), BRT technique (2D or 3D), concurrent chemotherapy (single agent or double agent), concurrent chemotherapy cycles, neoadjuvant chemotherapy (yes or no), consolidate chemotherapy (yes or no), progression status and surviving status were collected. In this study, EBRT was typically delivered in 3 modes: 45 Gy/25f, 50.4 Gy/28f, or 50 Gy/25f, and the Equivalent Dose in 2 Gy/f (EQD2) during EBRT (EBRTeqd2) of every patients would be calculated via the equation as follows:


EQD2=nd*(d+α/β)/(2+α/β).

In the equation, *n* was the number of fractions, d represented the dose in 1 fraction, and *α*/*β* = 10. Furthermore, the EQD2 of BRT (BRTeqd2) would also be calculated via the equation, and HRCTV-D90 and dosage point A were used for the calculation in 3D and 2D techniques, respectively. And the total EQD2 (Totaleqd2) of every patients was estimated by summing the EBRTeqd2 and BRTeqd2.

### Follow-up

The patients would receive follow-up as protocol: 1-2 months after the completion of CCRT would be the first follow-up time, the frequency of follow-up would be every 3 months for first 2 years, every 6 months for another 3-5 years, and annual follow-up after 5 years.^17^ The gynecological examination, abdominal ultrasonography, and MRI scanning were conducted to identify persistent and recurrent disease.

### Outcome

The 5-year overall survival (OS) and progression-free survival (PFS) would be set as the primary and secondary endpoint, respectively, where the OS was defined as the time from diagnosis to death from any cause or censor, and PFS was calculated as the time from diagnosis to any evidence of disease progression. Moreover, the survival differences would be compared between the presence of VI or not in each group to find out whether the presence of VI would further impair the survival probability of stage IIB CC patients.

### Statistical analysis

Categorical variables were counted as percentages or frequencies and compared using the Pearson χ^2^ test, continues variables were described as medians with IQR and compared with *t*-test. The 5-year PFS and OS were calculated and visualized by Kaplan–Meier method and log-rank test was performed to assess the difference in survival outcomes between 2 groups. And the survival difference between UL and BL groups were compared before and after propensity scoring matching (PSM) analysis, which was able to mitigate the impact of imbalanced confounding factors across the groups.^18^

The R package “randomForest” was implemented to conduct the random forest (RF) selection, where 100 was set as the number of Monte Carlo Iteration, and the features with relative importance greater than 0 would be identified. Accordingly, the included patients were divided into a training and validation cohort with a ratio of 7:3. The RF analysis was conducted in the training cohort to identify important factors associated with survival outcomes of stage IIB CC patients, and the feature importance of the factors in prognosis prediction was also ranked. The predicting performance of RF model would be assessed both in the training and validation cohort using the area under the receiver operating characteristic curve (AUC). Furthermore, the results from RF model were interpreted by the SHAP tool, which aided in giving insights into the process of black-box prediction in machine learning algorithm (MLA).^19^ In essential, this approach could quantify and prioritize the importance of each feature in the prediction output through estimating the SHAP value of each input features.

All analyses were performed using SPSS v24.0, R software (version.3.6.1; http://www.r-project.org), and an online statistical tool (https://www.xsmartanalysis.com) which was constructed based on Python (version 3.7.1). A 2-tailed *P* < 0.05 was considered as statistically significant.

## Results

### Patients characteristics

**Figure 1.**
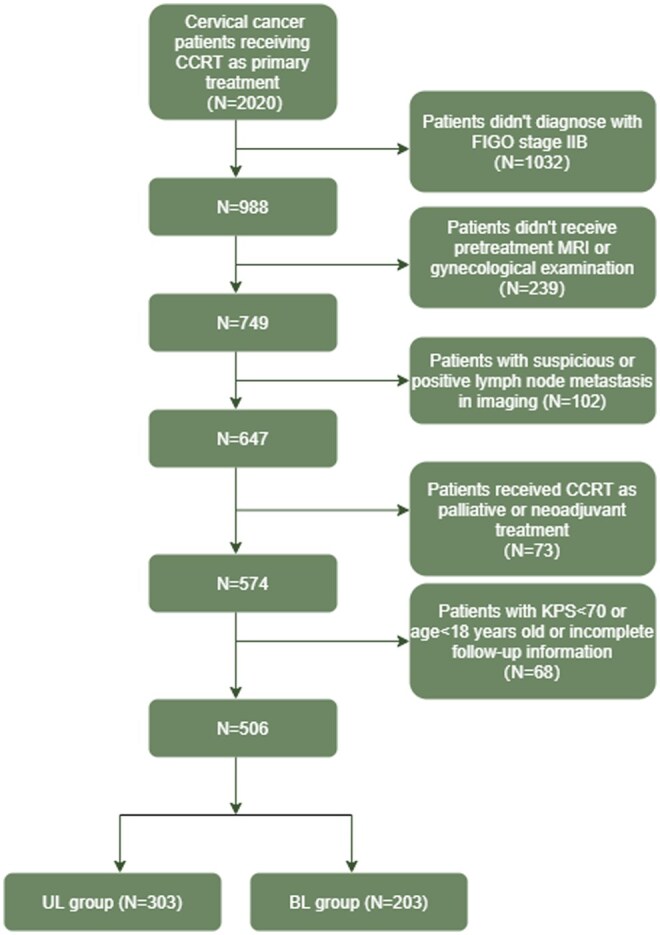
The detailed selection process of the included patients.

A total of 506 stage IIB CC patients were included in the final analysis, among whom 203 and 303 patients were assigned to BL and UL groups, respectively, after thorough review of their gynecologic examination and MRI scanning. Of note, the detailed process for selection was listed in Figure 1. The median follow-up time for the included patients was 86 months (IQR: 39-142). In the UL cohort, it was easy to notice that left side of PI was much more prevalent than the right side of PI (61.4% vs. 38.6%). Statistically significant difference in patients distribution between the BL and UL group could be observed in VI, EBRT technique, EBRT dose, BRT technique, and concurrent chemotherapy. Notably, more patients in the BL group developed VI than those in the UL group (70.4% vs. 58.4%, *P* = 0.008), which further highlighted the aggressiveness of BL disease. Besides, a larger proportion of patients in the BL group received the 3D BRT technique than that in UL group (45.3% vs. 28.7%, *P* < 0.001), which could be partially explained by the fact that more patients in the BL patients might need interstitial BRT during the treatment (Table 1).

**Table 1.** Demographic and clinico-pathological characteristics in BL and UL cohort.

Variables	**BL cohort (*N*** **=** **203)**	**UL cohort (*N*** **=** **303)**	*P*-Value
**Age**	53 (IQR: 47-58)	52 (IQR: 45-59)	0.860
**Vaginal invasion**			**0.008**
Yes	143 (70.4%)	177 (58.4%)	
No	60 (29.6%)	126 (41.6%)	
**Side of parametrial invasion**			
Right	203 (100%)	117 (38.6%)	
Left	203 (100%)	186 (61.4%)	
**Tumor size (cm)**	4 (IQR: 3.3-5)	3.8 (IQR: 2.5-5.5)	0.162
**Histology**			0.666
Squamous	186 (91.6%)	282 (93.1%)	
Non**-**squamous	17 (8.4%)	21 (6.9%)	
**EBRT technique**			**<0.001**
3D-CRT	103 (50.7%)	118 (38.9%)	
FF-IMRT	70 (34.5%)	87 (28.7%)	
VMAT	30 (14.8%)	98 (32.3%)	
**EBRT dose**			**<0.001**
45 Gy/25f	46 (22.7%)	51 (16.8%)	
50.4 Gy/28f	42 (20.7%)	125 (41.3%)	
50 Gy/25f	115 (56.6%)	127 (41.9%)	
**BRT technique**			**<0.001**
2D	111 (54.7%)	216 (71.3%)	
3D	92 (45.3%)	87 (28.7%)	
**BRT dose (EQD2)**	45.28 (IQR: 40-48)	45.1 (IQR: 40-48)	0.961
**Total dose (EQD2)**	88.5 (IQR: 81.8-94)	88.5 (IQR: 81.6-93.8)	0.879
**Concurrent chemotherapy**			**0.007**
Single agent	161 (79.3%)	268 (88.4%)	
Double agent	42 (20.7%)	35 (11.6%)	
**Concurrent chemotherapy cycles**			
Single agent	4 (IQR: 3-5)	4 (IQR: 4-5)	0.882
Double agent	2 (IQR: 1-2)	2 (IQR: 1-2)	0.995
**Neoadjuvant chemotherapy**			**0.338**
Yes	25 (12.3%)	28 (9.2%)	
No	178 (87.7%)	275 (90.8%)	
**Consolidate chemotherapy**			**0.791**
Yes	30 (14.8%)	41 (13.5%)	
No	173 (85.2%)	262 (86.5%)	
**Progression status**			**0.005**
Yes	68 (33.5%)	66 (21.8%)	
No	135 (66.5%)	237 (78.2%)	
**Death status**			**<0.001**
Yes	62 (30.5%)	41 (13.5%)	
No	141 (69.5%)	262 (86.5%)	

Abbreviations: 2D, two-dimensional; 3D, three-dimensional; 3D-CRT, three-dimensional conform radiotherapy; BL, bilateral parametrial invasion; BRT, brachytherapy; EBRT, external beam radiotherapy; IMAR, intensity modulated radiotherapy; UL, unilateral parametrial invasion; VAMT, volumetric modulated arc therapy. Bold values meant *P*<0.05.

### Survival outcomes

**Figure 2.**
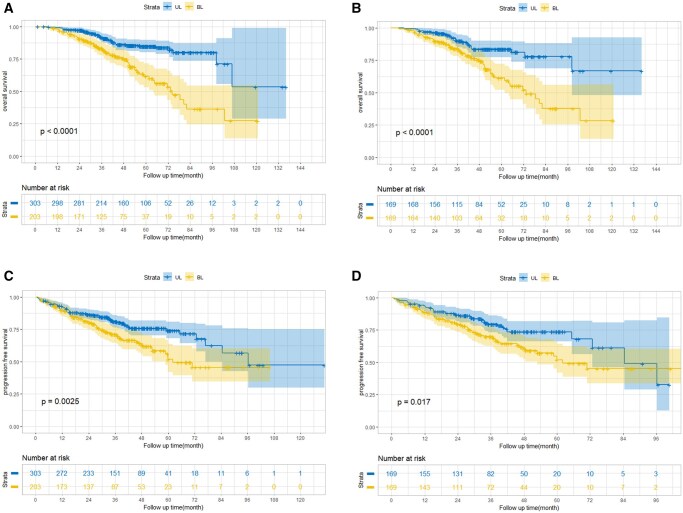
The comparison of OS between UL and BL before (A) and after (B) the PSM; The comparison of PFS between UL and BL before (C) and after (D) the PSM. Of note, VI, EBRT technique, EBRT dose, BRT technique, and concurrent chemotherapy were incorporated into the PSM process. Abbreviations: BL, bilateral parametrial invasion; BRT, brachytherapy; EBRT, external body radiotherapy; OS, overall survival; PFS, progression-free survival; PSM, propensity score matching; UL, unilateral parametrial invasion; VI, vaginal invasion.

The 5-year OS rate in the BL and UL group was 61.7% (95% CI: 57.0-66.4) and 84.8% (95% CI: 82.4-87.2), respectively before PSM (HR = 2.83, 95% CI: 1.90-4.20, *P* < 0.001) (Figure 2A). After PSM, the 5-year OS rate was 61.3% (95% CI: 56.6-66.0) in the BL group, while that in UL group was 81.2% (95% CI: 77.3-85.1) (HR = 2.51, 95% CI: 1.56-4.04, *P* < 0.001) (Figure 2B). Similarly, patients in UL group have longer PFS than that in the BL group, whether before or after PSM. In detail, the 5-year PFS rate in BL group was 51.8% (95% CI: 47.0-56.6) while that in UL group was 74.1% (95% CI: 70.9-77.3) (HR: 1.68, 95% CI: 1.19-2.35, *P* = 0.0025) before PSM (Figure 2C). After PSM, the 5-year PFS was 49.3% (95% CI: 45.4-53.2) and 73.6% (95% CI: 69.7-77.5) in BL and UL groups, respectively (HR: 1.62, 95% CI: 1.09-2.43, *P* = 0.017) (Figure 2D). Moreover, we compared the survival differences between stage IIB CC patients with and without the presence of VI to explore the impact of VI status on the survival outcomes for these patients. Statistically significant differences could be observed between UL and BL group while survival differences between different VI status was not that obvious (Supplementary Figure S1). Specifically, the 5-year OS was 85.5% (95% CI: 82.4-88.6) and 83.7% (95% CI: 80.1-87.3) in UL-VI and UL+VI group, respectively (HR: 0.70, 95% CI: 0.30-1.66, *P* = 0.85) (Supplementary Figure S2a). Similarly, the 5-year OS was 52.9% (95% CI: 49.3-56.5) in BL-VI and 45.8% (95% CI: 41.9-49.7) in BL + VI group (HR: 0.95, 95% CI: 0.58-1.54, *P* = 0.49) (Supplementary Figure S2c). Seemingly, the presence of VI would not significantly impair the survival outcomes for stage IIB CC patients, which could be further verified in PFS (Supplementary Figure S2b and d).

The recurrence pattern between the UL and BL group as further compared, which showed that the most common recurrence pattern in both groups was distant metastasis (63.9% vs. 64.3%), and no significant difference regarding recurrence pattern between 2 groups was observed (*P* = 0.739) (Supplementary Table S2). However, a bigger proportion of patients in BL group were inclined to develop distant metastasis than those in UL group, with distant metastasis occurred in 15.3% and 7.6% patients in BL and UL groups, respectively (*P* = 0.004) (Supplementary Figure S3), which might imply that additional treatment like consolidate chemotherapy could be an option for patients with BL after the completion of CCRT.

### RF model development and performance

**Figure 3.**
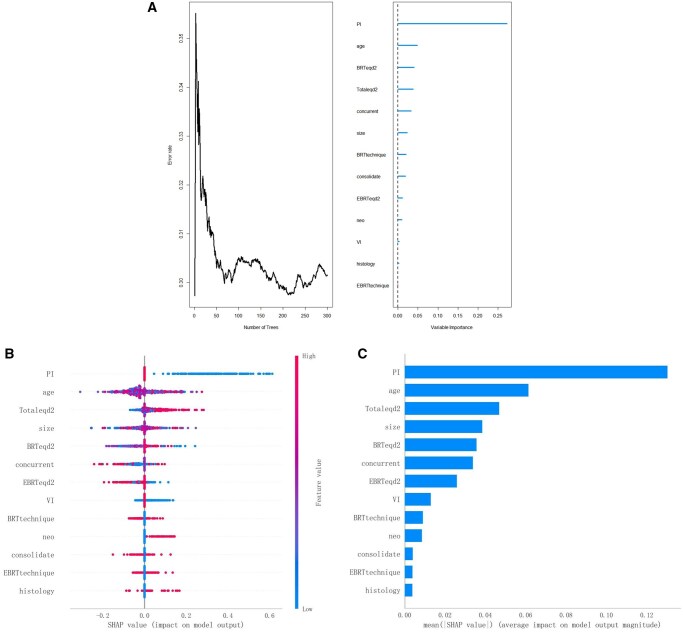
The generating process of the RF model, where the prediction error rate is located between 0.30 and 0.31, as the increasing of trees number. Also, the ranking of feature importance of variables in the model was depicted (A). The Beeswarm summary plot from SHAP analysis was designed to exhibit the impact of top features on RF model output (B). The mean SHAP value of each feature was displayed via bar chart, which showed the average impact of each feature on the model output (C). Abbreviations: RF, random forest; SHAP, SHapley Additive exPlanation.

The total cohort with 506 patients was divided into a training and a validation cohort with 354 and 154 patients in each cohort, respectively. No significant difference was found between the training and validation cohort (All *P* > 0.05) (Supplementary Table S3). The “randomForest” package was used during the construction of the RF model. All clinical features were incorporated into the development of the RF model, which showed that the error rate stabilized between 0.30 and 0.31 as the number of trees increased. The importance of every feature in predicting the OS was ranked and listed, where PI was the most important variable in predicting OS (Figure 3A). Furthermore, the predicting performance of RF model was evaluated, which showed decent ability in predicting OS for stage IIB CC patients with AUC of 0.89 (95% CI: 0.87-0.91) and 0.88 (95% CI: 0.80-0.96) in the training and validation cohort, respectively (Supplementary Figure S4).

### The interpretation of model

The SHAP was introduced in this study to better interpret the contribution of each predictor from RF model in predicting OS for stage IIB CC patients. The importance of each predictor was ranked in descending order, which was comparable with the results of RF model (Figure 3A,B). Strikingly, PI demonstrated with the greatest predictive ability in the model, followed closely by age and Totaleqd2 (Figure 3B). Moreover, the Figure 3B illustrated how each predictor influences the survival outcome, which showed that the PI was negatively related to survival probability, where BL was assigned with feature value of “1” and UL with “0,” and the feature regarding PI negatively contributed to survival with a bigger feature value. While radiation dose including Totaleqd2 and BRTeqd2 positively contributed to OS. Besides, neoadjuvant and consolidate chemotherapy were also observed to be positively associated with survival outcome though their importance ranking was not high (Figure 3B). Concurrent chemotherapy ranked much higher than neoadjuvant and consolidate chemotherapy, which to some extent highlighted the important role of CCRT in treating stage IIB CC patients. Furthermore, an inclusive SHAP value could be generated for each predictor, which was aid in quantifying the contribution of each predictor making for the output of model. Figure 3C measured the average SHAP value of each predictor with the length of the bar, which showed that PI was approximately 1 time longer (ie, more important) than age and Totaleqd2 and further underpinned the paramount role PI has played in predicting the survival outcomes for stage IIB CC patients (Figure 3C).

## Discussion

In the current study, we assessed the role of UL and BL in affecting the survival for 2018 FIGO stage IIB CC patients undergoing CCRT as primary treatment, which demonstrated that patients with BL had worse survival outcomes compared to patients with UL. Furthermore, RF model and SHAP analysis were performed to reveal the paramount importance of UL and BL in contributing to the survival outcomes among stage IIB CC patients. In addition, consolidated chemotherapy might be an alternative for the enhancement of treatment for patients with BL.

A few previous studies have made their attempt to identify the predictors associated with survival outcomes among stage IIB CC patients, while patients with LN metastasis were not excluded from these studies and some included patients received surgery as primary treatment, which was not considered as standard treatment for these patients.^11–14^ Therefore, reappraising the factors associated with stage IIB CC patients in the 2018 FIGO staging era was still necessary.

Although factors like age, histology, and tumor size have been evaluated in prognosis prediction among stage IIB CC patients, only 1 historical study conducted in 1992 assessed the prognostic significance of PI for these patients.^15^ A total of 413 stage IIB CC patients were included in the 1992 study, where patients with UL disease performed better than those with BL disease in 4-year OS (70% vs. 52%, *P* = 0.001) and 4-year in-field pelvic control (83% vs. 69%, *P* = 0.001).^15^ Although this study highlighted the significant role of UL and BL in differentiating the survival outcomes among stage IIB CC patients, it was worth noting that the inclusion of patients with LN metastasis and less advanced radiation technique used during that period would impair the credibility and applicability of the results. In order to avoid these shortcomings, we conducted this multi-center study including only 2018 FIGO stage IIB CC patients treated with a more advanced radiation technique. Intriguingly, stage IIB CC patients with UL disease outperformed those with BL disease both in OS and PFS. Moreover, this conclusion was still solid even after all baseline characteristics had been balanced, which was consistent to the 1992 study. But we could also observed that the survival outcomes of the 1992 study were inferior to our study among stage IIB CC patients with UL and BL disease, respectively, which could be partially explained by the inclusion of patients with LN metastasis and less advanced radiation technique used in 1992 study.

In our study, we further evaluated the importance of baseline characteristics in predicting the survival outcomes for stage IIB CC patients by performing the RF model, which was able to select the significant factors affecting the outcomes and rank the importance of incorporating features in prognosis prediction.^20^ All included features except the EBRT technique were found to contribute to the survival outcomes, which showed that, in terms of treatment efficacy, the IMRT technique might not be superior to 3D-CRT in treating stage IIB CC patients. Our result was in line with established literature showing that equivalent survival outcomes could be obtained from 3D-CRT or IMRT radiation technique for CC patients, while gastrointestinal (GI) and genitourinary (GU) toxicity were significantly reduced among patients receiving IMRT technique, which supported the widespread promotion of IMRT technique for the treatment of CC patients in recent clinical practice.^21^^,^^22^ Moreover, our findings from the RF algorithm indicated that the paramount feature in predicting survival outcomes among stage IIB CC patients was PI, followed by patients’ age and radiation dose. Although previous researches have established the role of features including age, size, and radiation dose in prognosis prediction,^11–13^^,^^23^ our team might be the first 1 to rank the feature importance in predicting the prognosis for stage IIB CC patients using MLA. Besides, the SHAP was introduced in our study to demystify the RF model and avoid potential opacity and bias caused by black-box nature of many MLAs, which further hindered their widespread acceptance in daily clinical practice.^24^ According to our findings, PI ranked first out of thirteen features affecting the survival outcomes among stage IIB CC patients, and the Beeswarm plot also demonstrated that PI was negatively related to the survival. Furthermore, the SHAP value quantifying the feature importance was estimated and visualized through bar chart, which revealed that PI was much more important in predicting prognosis than other selected features. Although the uniformity rate between pathological PI and gynecological examination PI was modest (56.0%; 95% CI: 52.4%-59.7%),^11^ we tried to improve the rate in our study by identifying stage IIB CC patients through combining the gynecological examination and MRI scanning, which, to some extent, increased the reliability of the stage of included patients.

We further appraised the role of VI, categorized as stage IIA according to the 2018 FIGO staging system, has played in stage IIB CC patients. And our findings indicated that the presence of VI wouldn’t deteriorate the prognosis for stage IIB CC patients, irrespective of patients with UL or BL disease. In contrast, the study conducted by Fang et al found that stage IIIB CC patients with lower third vaginal involvement had worse survival than those without.^25^ A plausible explanation for this might be the greater chance for patients with lower third vaginal involvement to develop inguinal LN metastasis. Nevertheless, the chance of developing LN metastasis among patients with PI was much bigger than those with VI, which partially clarified why the presence of VI didn’t significantly impact the survival outcomes among stage IIB CC patients.

As to the significance of chemotherapy, our findings strengthened the role of concurrent chemotherapy being cornerstone for the treatment of stage IIB CC patients as recommended by the National Comprehensive Cancer Network (NCCN) guidelines.^9^ On the contrary, neoadjuvant and consolidate chemotherapy were not as important as concurrent chemotherapy, which ranked sixth in feature importance according to SHAP, while neoadjuvant and consolidate chemotherapy were listed as 10th and 11th important features, respectively. Two randomized controlled trials (RCTs) have assessed the role of consolidate chemotherapy in the treatment of LACC patients and demonstrated that consolidated chemotherapy after CCRT wouldn’t bring additional survival benefit when compared to CCRT alone,^26^^,^^27^ which was further verified in our study showing that the bar representing concurrent chemotherapy was approximately eight times longer than that representing consolidate chemotherapy (Figure 2C), suggesting that concurrent chemotherapy was much more important treatment modality for stage IIB CC patients. However, the value of consolidated chemotherapy in patients with BL disease might need future assessment, considering that the feature importance was ranked among stage IIB patients, including both the UL and BL patients.

In this study, sophisticated MLA was first utilized to quantify the feature importance in contributing to the survival outcomes among stage IIB CC patients, and SHAP analysis was further performed to explain the results derived from the MLA and increased the interpretability and applicability of the results. As a result, PI stood out to be the paramount feature associated with the prognosis, demonstrating that patients with UL disease outperformed those with BL disease in prognosis irrespective of the status of VI. Besides, the multi-center design nature of this study would further avoid potential selection bias and increase the credibility and generalizability of our findings. However, some limitations of this study can’t be overlooked. First, although the pretreatment gynecological examination was performed by experienced gynecological oncologists in this study, the judgment and recognition toward PI varied from doctor to doctor. Second, some established features, including SII and squamous cell carcinoma antigen (SCC Ag) were not collected and analyzed in our study. Third, only 7.5% of included patients were diagnosed with non-squamous disease in this study, which might impair the reproducibility of the feature importance ranking of histology. Moreover, RF model used to identify the important feature was trained and internally validated in this study, while external validation might needed to further increase the generalizability of the conclusion. Last, stage IIB patients receiving surgery as initial treatment were not included in this study, though some previous studies have shown similar survival outcomes between surgery and CCRT.^28^^,^^29^ therefore, the survival difference between UL and BL might be worth of further investigation in stage IIB CC patients receiving neoadjuvant chemotherapy and surgery.

## Conclusion

A multi-center study was conducted to assess potential features associated with the prognosis among 2018 FIGO stage IIB CC patients, where PI was found to be the most important feature in predicting prognosis for these patients, and patients with UL disease performed much better than those with BL disease both in OS and PFS. Therefore, the UL or BL disease among patients with PI might be taken into consideration in the following revision of the FIGO staging system. Furthermore, the presence of VI didn’t significantly impact the prognosis for stage IIB CC patients, and patients with or without VI had comparable survival whether in the UL or BL group. Few of patients with stage IIB CC would receive surgery given that the recommended treatment for those patients was CCRT; thus, further improving the diagnostic accuracy would be crucial for estimating the survival probability and identifying predicting features for stage IIB CC patients.

## Supplementary Material

oyaf329_Supplementary_Data

## Data Availability

All data that support the findings of this study are available from the corresponding author upon reasonable request.
