# Supplementary material for: Different prognosis for stage IIB cervical cancer patients with unilateral or bilateral parametrial invasion treated with concurrent chemoradiotherapy
Source: Oncologist. 2025 Oct 16;30(11):oyaf329. doi: 10.1093/oncolo/oyaf329 (PMC12852999; doi:10.1093/oncolo/oyaf329)
Supplement: oyaf329_Supplementary_Data [file oyaf329_supplementary_data.zip › supp.R2.docx]

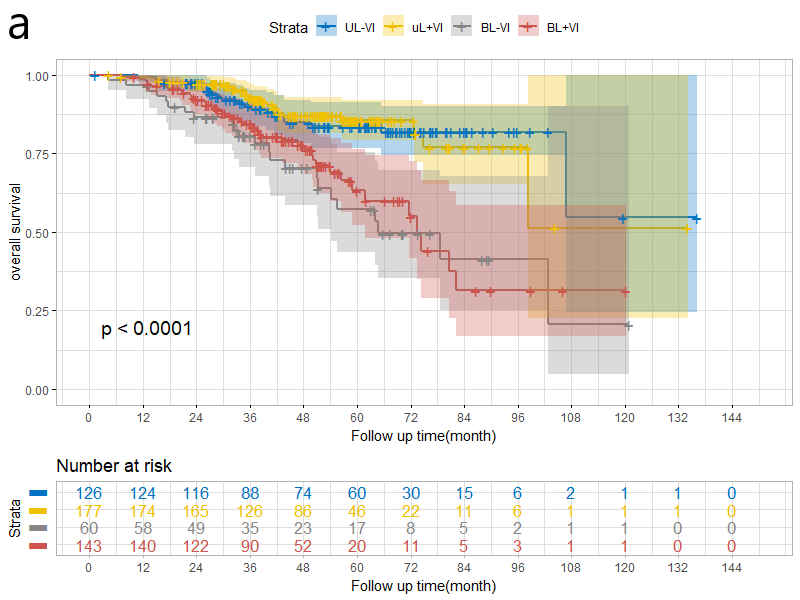

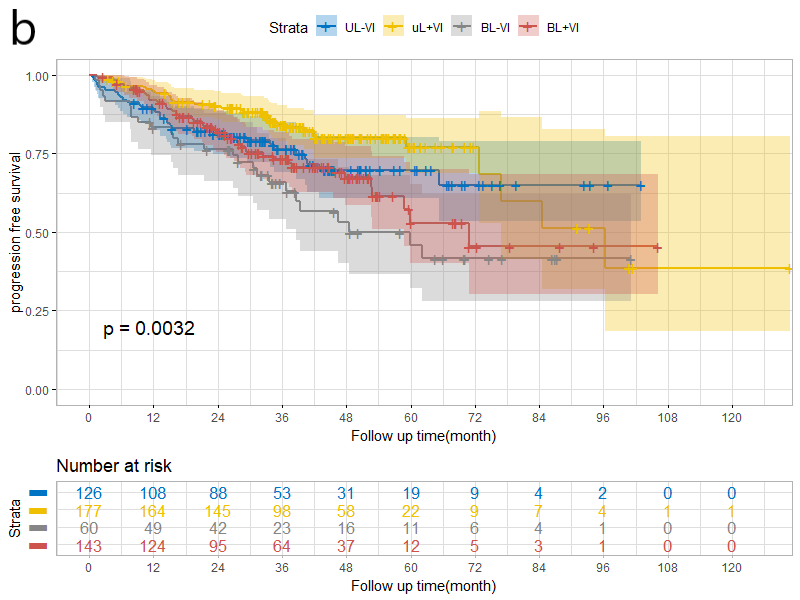


**Fig.S1** The comparison of OS (a) and PFS (b) between unilateral parametrial invasion without vaginal invasion (UL), unilateral parametrial invasion plus vaginal invasion (UL+VI), bilateral parametrial invasion without vaginal invasion (BL) and bilateral parametrial invasion plus vaginal invasion (BL+VI).


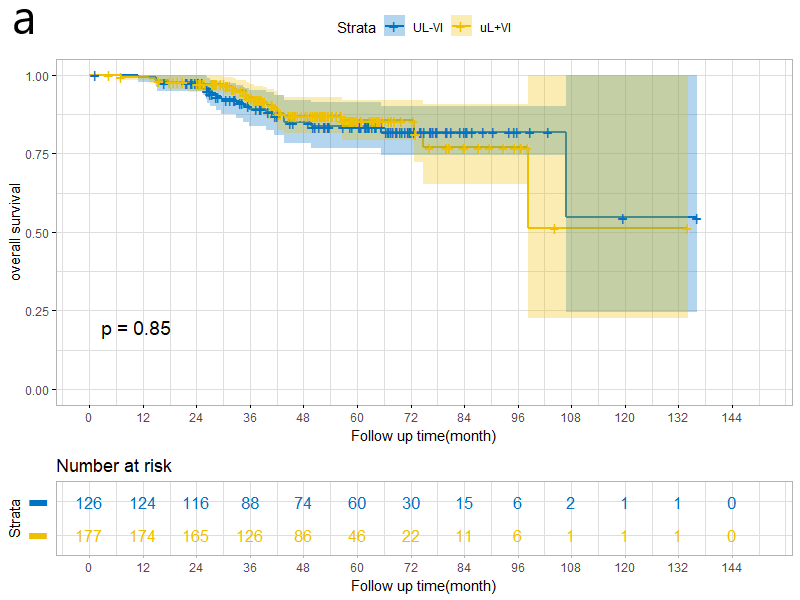

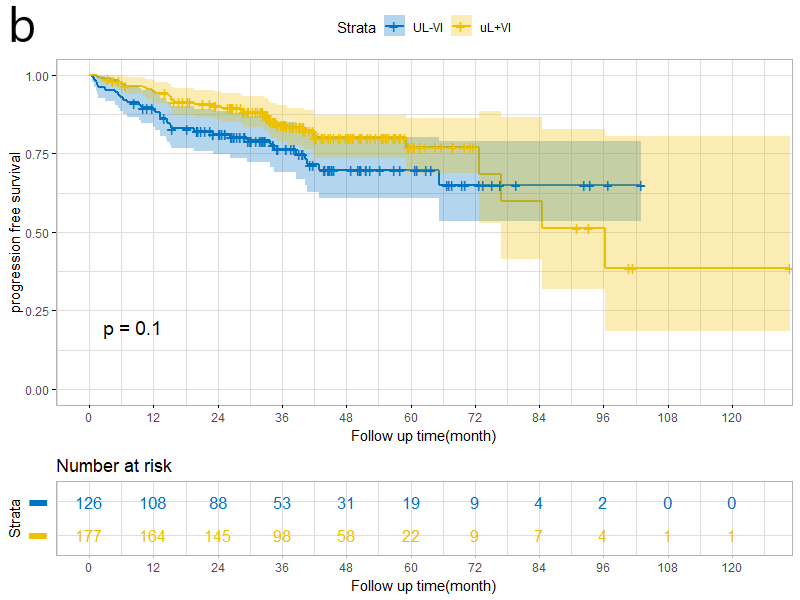


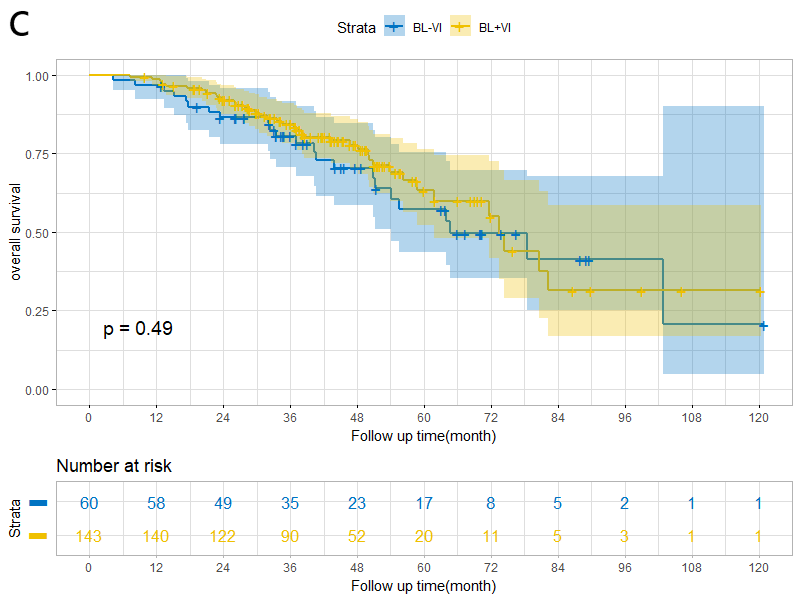

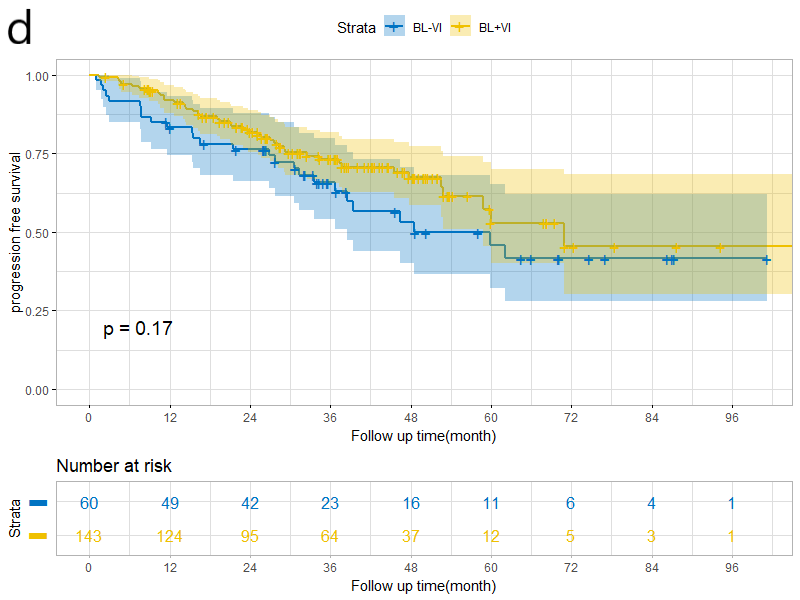


**Fig.S2** The comparison of OS (a) and PFS (b) between unilateral parametrial invasion without vaginal invasion (UL-VI) and unilateral parametrial invasion plus vaginal invasion (UL+VI); The comparison of OS (c) and PFS (d) between bilateral parametrial invasion without vaginal invasion (BL-VI) and bilateral parametrial invasion plus vaginal invasion (BL+VI).


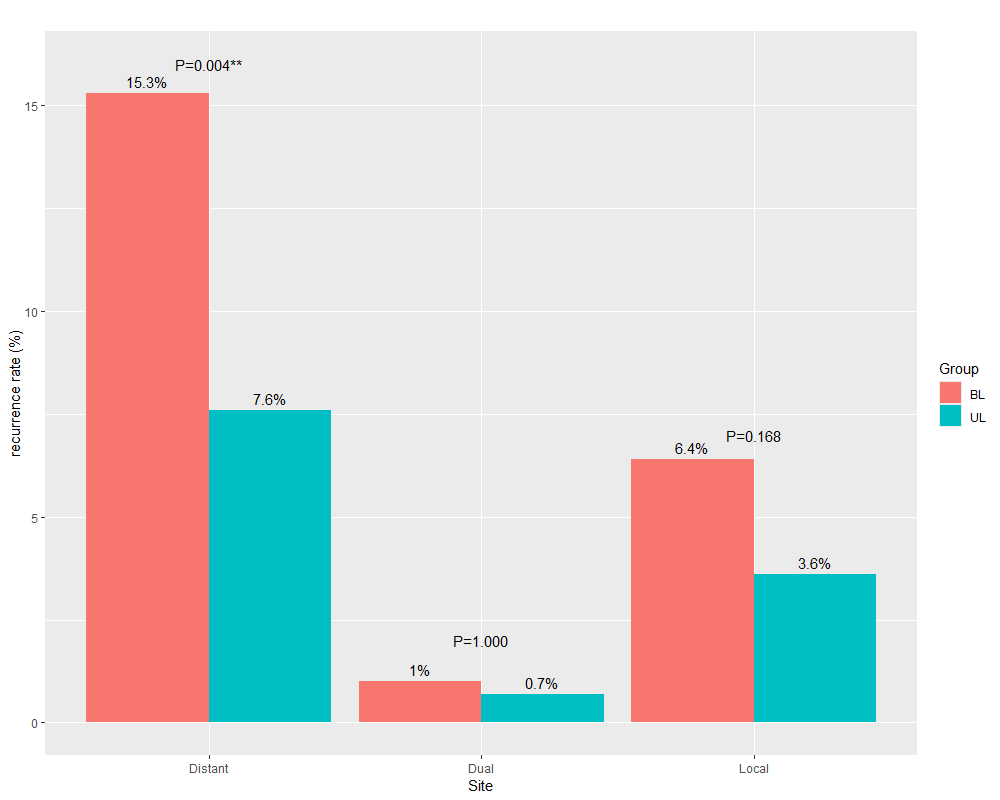


**Fig.S3** The comparison of recurrence rate of distant area, local area and dual area between bilateral parametrial invasion (BL) and unilateral parametrial invasion (UL) group.


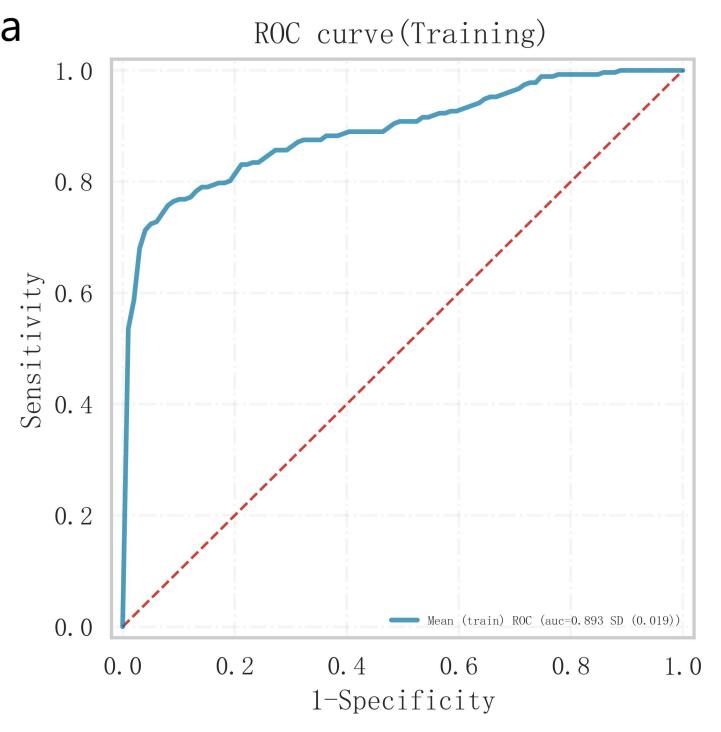

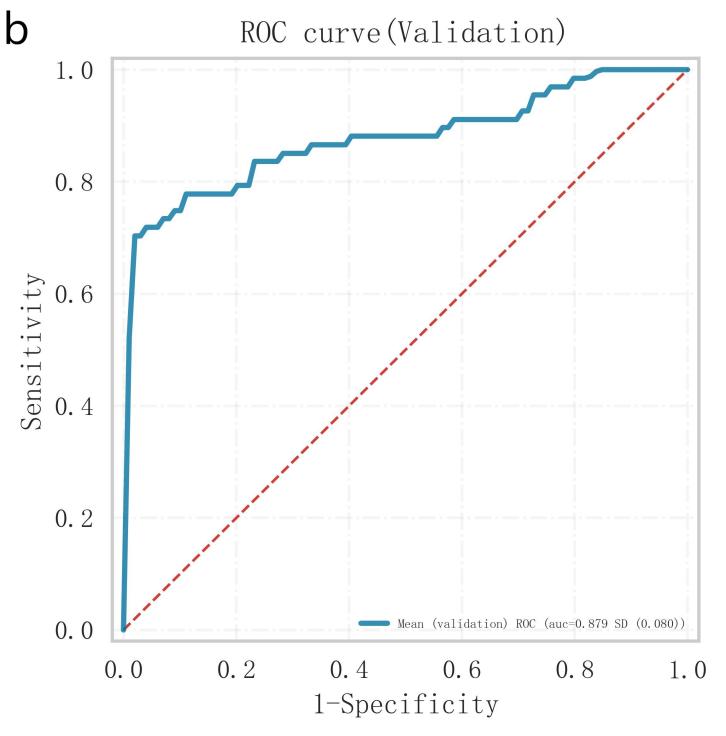


**Fig.S4** Depicting ROC curves to represent the AUCs of the random forest (RF) model both in the training (a) and validation cohort (b).

| **Table.S1 The IRB number from each research center** | |
| --- | --- |
| **Hospital** | **IRB number** |
| Peking Union Medical College Hospital | S-K140 |
| Xijing Hospital | XJH-2015-0139 |
| General Hospital of Ningxia Medical University | NK-068 |
| The Second Affiliated Hospital of Jilin University | JU-L190 |
| Affiliated Cancer Hospital of Guizhou Medical University | GMUCH-2015-087 |
| The Second Affiliated Hospital of Dalian Medical University | DLYK-2015055 |
| Affiliated Hospital of Inner Mongolia Medical University | IMM-L23 |
| Peking University First Hospital | PUFH-K098 |
| First Affiliated Hospital of Xian Jiaotong University | XAJT-NK-131 |
| The First Affiliated Hospital of Zhengzhou University | ZZU-LK-099 |
| The 940th Hospital of Joint Logistics Support Force of Chinese People's Liberation Army | PLA-JK-091 |
| Gansu Province Cancer Hospital | GSZL-2015-036 |
| Guangxi Medical University Cancer Hospital, 71 Hedi Road, Qingxiu District | GXMUCH-087 |
| The Affiliated Cancer Hospital of Xinjiang Medical University | XJMUCH-KK-077 |
| China-Japan Union Hospital of Jilin University | CJUH-AK-076 |
| Cangzhou Central Hospital | CZCH-LL-012 |
| Jilin Province Cancer Hospital | JPCH-2015-072 |

| **Table.S2** Comparison of the recurrence pattern between UL and BL group. | | | |
| --- | --- | --- | --- |
|  | **UL (N=36)** | **BL (N=46)** | ***P* value** |
| **Failure region** |  |  | 0.739 |
| Local | 11 (30.6%) | 13 (28.3%) |  |
| Distant | 23(63.9%) | 31 (67.4%) |  |
| Dual | 2 (5.5%) | 2 (4.3%) |  |

BL:Bilateral parametrial invasion; UL:Unilateral parametrial invasion.

| **Table.S3** Demographic and clinico-pathological characteristics in training and validation cohort. | | | |
| --- | --- | --- | --- |
| **Variables** | **Training cohort (N=354)** | **Validation cohort (N=152)** | ***P*-value** |
| **Age** | 52 (IQR: 46-58) | 52 (IQR: 46-57) | 0.988 |
| **Vaginal invasion** |  |  | 0.744 |
| Yes | 128 (36.2%) | 58 (38.2%) |  |
| No | 226 (63.8%) | 94 (61.8%) |  |
| **Parametrial invasion** |  |  | 0.918 |
| UL | 213 (60.2%) | 90 (59.2%) |  |
| BL | 141 (39.8%) | 62 (40.8%) |  |
| **Tumor size (cm)** | 4 (IQR: 2.9-4.6) | 4 (IQR: 3-5) | 0.739 |
| **Histology** |  |  | 1.000 |
| Squamous | 327 (92.4%) | 141 (92.8%) |  |
| Non-squamous | 27 (7.6%) | 11 (7.2%) |  |
| **EBRT technique** |  |  | 0.834 |
| 3D-CRT | 156 (44.1%) | 65 (42.8%) |  |
| FF-IMRT | 107 (30.2%) | 50 (32.9%) |  |
| VMAT | 91 (25.7%) | 37 (24.3%) |  |
| **EBRT dose** |  |  | 0.628 |
| 45Gy/25f | 65 (18.4%) | 32 (21.1%) |  |
| 50.4Gy/28f | 115 (32.5%) | 52 (34.2%) |  |
| 50Gy/25f | 174 (49.1%) | 68 (44.7%) |  |
| **BRT technique** |  |  | 0.797 |
| 2D | 227 (64.1%) | 100 (65.8%) |  |
| 3D | 127 (35.9%) | 52 (34.2%) |  |
| **BRT dose (EQD2)** | 45.1 (IQR: 40-48) | 45.9 (IQR: 40-48) | 0.878 |
| **Total dose (EQD2)** | 88.6 (IQR: 81.5-94) | 88.5 (IQR: 81.4-94.1) | 0.921 |
| **Concurrent chemotherapy** |  |  | 0.660 |
| Single agent | 298 (84.2%) | 131 (86.2%) |  |
| Double agent | 56 (15.8%) | 21 (13.8%) |  |
| **Concurrent chemotherapy cycles** |  |  |  |
| Single agent | 4 (IQR: 3-5) | 4 (IQR: 3-5) | 0.998 |
| Double agent | 2 (IQR: 1-2) | 2 (IQR: 1-2) | 0.995 |
| **Neoadjuvant chemotherapy** |  |  | 0.653 |
| Yes | 39 (11.0%) | 14 (9.2%) |  |
| No | 315 (89.0%) | 138 (90.8%) |  |
| **Consolidate chemotherapy** |  |  | 0.791 |
| Yes | 53 (15.0%) | 18 (11.8%) |  |
| No | 301 (85.0%) | 134 (88.2%) |  |

BL:Bilateral parametrial invasion; UL:Unilateral parametrial invasion.
